# Does Mutual Interference Affect the Feeding Rate of Aphidophagous Coccinellids? A Modeling Perspective

**DOI:** 10.1371/journal.pone.0146168

**Published:** 2016-01-12

**Authors:** Nikos E. Papanikolaou, Nikos Demiris, Panagiotis G. Milonas, Simon Preston, Theodore Kypraios

**Affiliations:** 1 Department of Entomology and Agricultural Zoology, Benaki Phytopathological Institute, Athens, Greece; 2 Department of Statistics, Athens University of Economics and Business, Athens, Greece; 3 School of Mathematical Sciences, University of Nottingham, Nottingham, United Kingdom; Shanxi University, CHINA

## Abstract

Mutual interference involves direct interactions between individuals of the same species that may alter their foraging success. Larvae of aphidophagous coccinellids typically stay within a patch during their lifetime, displaying remarkable aggregation to their prey. Thus, as larvae are exposed to each other, frequent encounters may affect their foraging success. A study was initiated in order to determine the effect of mutual interference in the coccinellids’ feeding rate. One to four 4^th^ larval instars of the fourteen-spotted ladybird beetle *Propylea quatuordecimpunctata* were exposed for 6 hours into plastic containers with different densities of the black bean aphid, *Aphis fabae*, on potted *Vicia faba* plants. The data were used to fit a purely prey-dependent Holling type II model and its alternatives which account for interference competition and have thus far been underutilized, i.e. the Beddington-DeAngelis, the Crowley-Martin and a modified Hassell-Varley model. The Crowley-Martin mechanistic model appeared to be slightly better among the competing models. The results showed that although the feeding rate became approximately independent of predator density at high prey density, some predator dependence in the coccinellid’s functional response was observed at the low prey—high predator density combination. It appears that at low prey densities, digestion breaks are negligible so that the predators do waste time interfering with each other, whereas at high prey densities time loss during digestion breaks may fully accommodate the cost of interference, so that the time cost may be negligible.

## Introduction

Predator-prey systems are characterized by intricacy, driven by the interactions among individuals. Interactions of this kind can become more complex in environments where competition is intensive. In terms of feeding rate, competition is a density-dependent process which allows for indirect and direct interactions between foraging individuals, and thus it can be divided in two discrete phenomena [1]: exploitation competition, where individuals are affected by the amount of remaining resource which has been exploited by others and therefore depleted, and interference competition that occurs via direct interactions between foraging individuals. Furthermore, mutual interference involves individuals of the same species [2].

Modeling functional responses, i.e. the number of prey attacked per predator as a function of prey density [3], is a central goal for ecologists, since the assessment of populations performance allows for further decisions in environmental management. Following Holling [4], density-independent predation occurs via a continuous linear response to increasing prey density or when consumption rate does not further increase once a threshold prey density has been reached, where density-dependence occurs via a decelerating (type II) or sigmoid (type III) response. Ecologists are often time utilizing functional response models in a theoretical or applied basis in order to extract information on predators’ feeding behavior or modeling predator-prey dynamics (e.g. [5–8]). Although purely prey-dependent functional response models are predominant in the literature and are mainly based on Holling’s disc equation [6], theoretical and empirical studies support the importance of predator density in functional responses [2, 9, 10]. Incorporating behavioral interactions between foraging predators, several modeling approaches have been conducted that account for interference competition, based on phenomenological [11] or mechanistic [12, 13, 14] approaches. These studies suggest that predation is not only prey-dependent but also a predator-dependent process, accounting for interference effects in the predators’ feeding rate. However, the application of these models is underutilized so far in the ecological literature, probably due to their complexity when compared to the simpler disc equation. Thus, there is only little available information on their viability (e.g. [9, 10]).

Predaceous coccinellidae species (Coleoptera: Coccinellidae) are established predators of several insect pests, such as aphids and coccids [15]. Their ability as biocontrol agents, as well as their abundance in different habitats, renders the careful study of these taxa imperative. Aphidophagous species are widely distributed in both Palearctic and Nearctic region, preying on numerous economically important aphid species (Homoptera: Aphididae) [16]. Their efficiency in suppressing aphids’ populations makes them popular in biological control, especially in short term periods [17]. Ecology of aphidophagous coccinellids suggests that they display aggregation to their prey [16, 18]. Additionally, larvae typically stay within a patch during their life, unlike adults, which are characterized by their ability to make flights [19, 20]. As larvae are exposed to each other, frequent encounters between individuals may affect their foraging success.

Given the importance of functional responses as a crucial component in community ecology, linking food webs and determining predator-prey dynamics [21], the selection of a functional response model that accurately describes predation seems indispensable. Here, we explore suggested functional response models that account for interference competition and are alternatives to Holling’s disc equation [9]. Our analysis concerned the larvae of a common predatory insect, the fourteen-spotted ladybird beetle *Propylea quatuordecimpunctata* L. (Coleoptera: Coccinellidae), which is widespread throughout Europe [16] and has been established in Nearctic region [22]. In addition to the ecological interpretation, we are interested in utilizing Bayesian inference in order to quantify the uncertainty of our estimates in a coherent, probabilistic manner. Recently, Papanikolaou et al. [23] suggested a Bayesian framework using Markov Chain Monte Carlo methods for fitting functional response models. Here we extend this approach in exploring more complex models.

## Materials and Methods

An original strain of *P*. *quatuordecimpunctata* was collected from *Zea mays* L. plants in Arta County (Northwestern Greece). Adults and immature stages were transferred to Biological Control Laboratory, Benaki Phytopathological Institute, and reared in Plexiglass cages (50 cm length × 30 cm diameter) at 25 ± 1°C, 65 ± 2% relative humidity and a photoperiod of 16L:8D. A stock colony of *Aphis fabae* Scopoli (Homoptera: Aphididae), fed on potted *Vicia faba* L. plants and maintained at 20 ± 1°C, was used as prey for the coccinellid.

There are numerous cases in which larvae (and/or eggs) of aphidophagous coccinellids are released in several habitats as biological control agents (e.g. [24–26]). This method serves as a useful tool in the conservation of coccinellids in agricultural habitats preventing migration. Thus, it is likely for the larvae during their lifetime to be exposed in small patches with several aphid densities. In this task, functional response experiments were conducted, where the experimental arena consisted of a single patch, i.e. a plastic container (12 cm height × 7 cm diameter) with a potted *V*. *faba* plant (at 8–9 cm height) bearing different densities of *A*. *fabae* (3–3.5 day-old, immature aphids to avoid any reproduction during the experiments). Newly emerged instars of *P*. *quatuordecimpunctata*, collected from the stock colony, were kept individually in plastic cages (5 cm height × 10 cm diameter) and fed ad libitum with instars and adults of *A*. *fabae*. We used 0.5–1.5 day-old fourth instars larvae. The prey densities tested were 5, 10, 15, 20 and 25 aphid nymphs for individual predators, 10, 20, 30, 40 and 50 nymphs for two predators, 15, 30, 45, 60 and 75 nymphs when predator density was three larvae and 20, 40, 60, 80 and 100 nymphs when predator density was four larvae. Exposure time was 6 h. We did not test higher predator densities, as it could result to larvae crowding and/or cannibalism which is common in laboratory-reared coccinellids, especially in low prey densities [27]. Ten replicates on each prey density were formed. At the end of each experimental period the number of living aphids was counted. The experiments were carried out at 25 ± 1°C, 65 ± 2% relative humidity, with a photoperiod of 16L:8D h.

A preliminary analysis was conducted using a logistic regression framework, in order to determine the shape of the functional response [28]. The results indicated a type II functional response for all datasets. Therefore, all the testing models were of this type of functional response. As ecological modeling is often based on Holling type II model, generally thought as a “null model” [7], the selected models are interesting alternatives thereof [9].

The mechanistic approach of Holling in type II functional response resulted in a prey-dependent model [29] of the form:

$$\frac{dN}{dt}=-\frac{aNP}{1+aT_{h}N}$$

where *N* denotes the prey density; *P* the predator density; *a* the predator’s attack rate, i.e. the per capita prey mortality at low prey densities and *T*_*h*_ the handling time which reflects the time, a predator spends on pursuing, subduing, eating and digesting its prey. An alternative of purely prey-dependent Holling’s modeling approach was presented by Hassell and Varley [11] and modified by Sutherland [30], assuming also predator-dependence in functional response, depending on the value of the *m* parameter:

$$\frac{dN}{dt}=-\frac{aNP^{\;m}}{1+aT_{h}NP^{\;m}}$$

where *m* denotes the magnitude of interference competition between predators. The Hassell-Varley model is thought of as a model of phenomenological nature. Beddington [12] and DeAngelis et al. [13] also considered Holling’s purely prey-dependent assumption and developed a mechanistic model commonly known as Beddington-DeAngelis model, which is an extension of Holling’s mechanistic approach:

$$\frac{dN}{dt}=-\frac{aNP}{1+aT_{h}N+bt_{w}\left(P-1\right)}.$$

Here *b* denotes the rate of encounter of a single predator with other predators and *t*_*w*_ the time wasted on an encounter. In its initial form, the DeAngelis model used *P* instead *P-1* in the denominator. Nevertheless, as a predator cannot encounter itself, the model is commonly used in the present form. Based upon the Beddington-DeAngelis approach, Crowley and Martin [14] developed a mechanistic predator-dependent model, known as Crowley-Martin model, which allows for interference between predators, which are also handling prey:

$$\frac{dN}{dt}=-\frac{aNP}{1+aT_{h}N+bt_{w}\left(P-1\right)+aT_{h}bt_{w}N\left(P-1\right)}.$$

When *P* = 1, the Hassell-Varley, Beddington-DeAngelis and Crowley-Martin models are equal to the Holling type II model.

Assuming a probability distribution, linking the data to the mechanistic assumption regarding the prey density, completes the model. The Gaussian and lognormal densities are often used for this purpose due to their simplicity. However, using continuous distributions when the responses (e.g. number of prey eaten) are discrete is not entirely satisfactory and can be potentially dangerous (e.g. [31]). Hence, we followed Papanikolaou et al. [23] in assuming a binomial distribution. We explicitly describe the model below: denote by *N*_*e*_(*t*) the number of prey eaten by time *t*. Since a prey item is either dead or alive by time *t* (which often denotes the end of the experiment), we assume that *N*_*e*_(*t*) follows a Binomial distribution with parameters *N*_0_ and *p*(*t*), where *N*_0_ is the initial prey population and *p*(*t*) is the probability that a prey item has been eaten by time *t*:

$$N_{e}\left(t\right)∼Binom\left(N_{0},p\left(t\right)\right)$$

$$p\left(t\right)=\left(N_{0}-N\left(t\right)\right)/\;N_{0}$$

where *N*_0_ is the initial number of prey and *N*(*t*) is given by the solution of the ordinary differential equation and evaluated at time *t*. Notice that none differential equations can be solved analytically and hence the solution has to be derived numerically. In addition, the solution of *N*(*t*) depends on each model’s parameter which we wish to infer from the data.

The parameters *b* and *t*_*w*_ are structurally non-identifiable since they always appear as a product. Hence, one cannot estimate both of those separately from the data. To this end, we grouped them into one parameter *c* which is a positive constant describing the magnitude of interference among predators [9, 10]. Hence, the Beddington-DeAngelis and Crowley-Martin models become:

$$\frac{dN}{dt}=-\frac{aNP}{1+aT_{h}N+c\left(P-1\right)}$$

and

$$\frac{dN}{dt}=-\frac{aNP}{1+aT_{h}N+c\left(P-1\right)+aT_{h}cN\left(P-1\right)}$$

respectively.

We adopted a Bayesian approach in which all the unknown model parameters are treated as random variables and the uncertainty of our estimates is quantified in a coherent, probabilistic manner. Within such a framework, a prior distribution is assigned to the parameters to express our prior beliefs about them before seeing the data. This information is then updated in the light of experimental data using Bayes theorem by multiplying it with the likelihood leading to the posterior distribution which contains all the information about the parameters. We used vague priors for most of the model parameters; typically, exponential distributions with very high variance (e.g. 1000000) to reflect our ignorance about them and allowed the posterior distribution of the parameters to be mostly informed by the data. However, we used the posterior distribution of the attack rate (angel) parameter, obtained from fitting the Holling model and by approximating it with a Gamma distribution (using the method of moments) as an informative prior for the more complex models, in order to overcome issues of practical non-identifiability. Our inference procedure was based upon Markov chain Monte Carlo methodology (e.g. [32]) that provides a generic framework for the accurate fitting of complex non-linear dynamic models to experimental data. In particular, we employed a random-walk Metropolis algorithm to draw samples from the posterior distribution of the parameters. The variance of the proposal distribution was tuned in order to achieve an acceptance rate of 25% [33]. The model determination process for models based upon ordinary differential equations is not a trivial matter. Roughly, there are two main types of methodology for model selection. The first is based upon Bayes factors and/or BIC and are true-model-consistent in the sense that, if the true model is among the entertained models, these methods will select the true model as more data arise. The second approach, based on AIC and/or cross-validation, dispenses with the need for the true model lying within the fitted models and selects the model with the best predicting performance. We opted for the latter group of techniques, since the mechanistic models we used are an ideal tool for giving an interpretable basis to our data but do not necessarily contain the "true" model. In the Bayesian framework it is advantageous to use the DIC [34] which performs the model selection process in a similar fashion to AIC but allows for the simultaneous estimation of model complexity.

## Results

**Fig 1:**
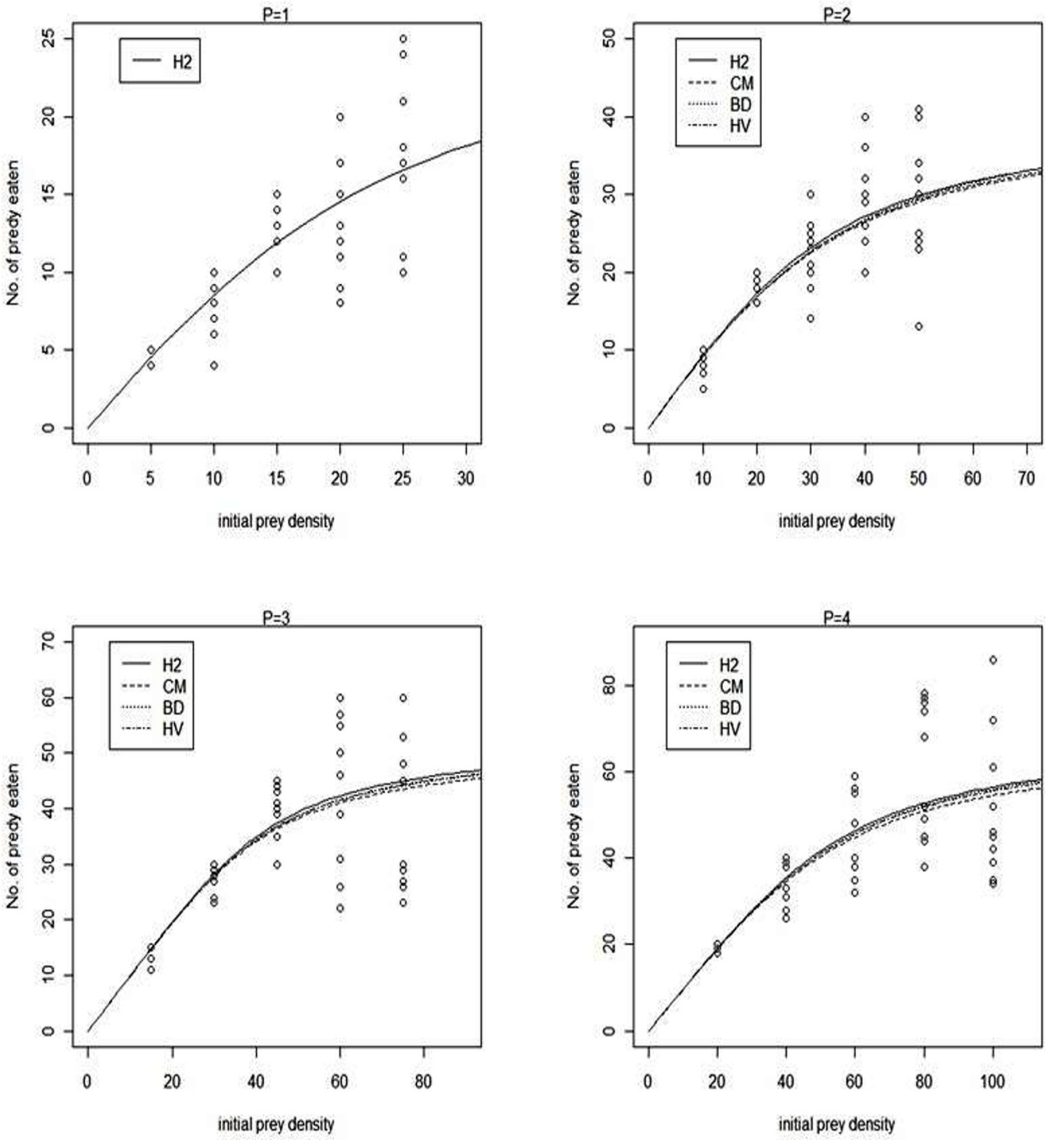
Observed versus fitted probabilities of *A*. *fabae* prey eaten by *Propylea quatuordecimpunctata*. Data fitted to Holling type II (H2,) Crowley-Martin (CM), Beddington-DeAngelis (BD) and Hassell-Varley (HV) models (*P* denotes the predator density).

The Holling type II, Crowley-Martin, Beddington-DeAngelis and Hassell-Varley models fitted the observed data reasonably well, as the fitted probabilities of the number of prey consumed lie within the main bulk of the data (Fig 1). The estimated parameters for each of the tested models, as well as the corresponding credible intervals, are presented in Table 1. In case of single predator -where all models are equivalent to Holling type II model- the predator’s mean handling time was 0.220h. As predator density increased, there was not statistically significant increase to the estimated handling times in all four tested models, indicating that predator per capita feeding rate is approximately independent of predator density at high prey density. The estimated mean handling times of Holling type II, Beddington-DeAngelis and Hassell-Varley models were very similar. Thus, when two, three and four predators were exposed the mean handling times were 0.293, 0.293 and 0.298, 0.342, 0.342 and 0.341, 0.343, 0.346 and 0.347 h for Holling type II, Beddington-DeAngelis and Hassell-Varley models, respectively, where the corresponding values of Crowley-Martin model were 0.249, 0.300 and 0.301 h.

**Table 1:** Estimated parameters (mean, 95% credible intervals) of the Holling type II (H2), Beddington-DeAngelis (BD), Crowley-Martin (CM) and Hassell- Varley (HV) models, fitted to *Propylea quatuordecimpunctata* functional response data.

parameter	*P = 1*	*P = 2*				*P = 3*				*P = 4*			
	H2	H2	BD	CM	HV	H2	BD	CM	HV	H2	BD	CM	HV
*a* (h^-1^)	0.464	0.287	0.320	0.315	0.324	0.340	0.381	0.364	0.371	0.184	0.208	0.203	0.207
	0.353–0.591	0.232–0.346	0.262–0.384	0.261–0.375	0.265–0.389	0.285–0.398	0.305–0.461	0.306–0.424	0.310–0.436	0.153–0.212	0.174–0.241	0.173–0.240	0.171–0.251
*T*_*h*_ (h)	0.220	0.293	0.293	0.249	0.298	0.342	0.342	0.300	0.341	0.343	0.346	0.301	0.347
	0.140–0.307	0.236–0.356	0.240–0.357	0.179–0.323	0.238–0.373	0.297–0.390	0.299–0.395	0.239–0.354	0.299–0.390	0.296–0.391	0.304–0.394	0.236–0.361	0.304–0.401
*c*	-	-	0.222	0.204	*-*	*-*	0.115	0.079	*-*	*-*	0.062	0.056	*-*
	-	-	0.017–0.579	0.011–0.530	*-*	*-*	0.005–0.314	0.004–0.204	*-*	*-*	0.005–0.152	0.003–0.142	*-*
*m*	-	*-*	*-*	-	0.283	*-*	*-*	*-*	0.147	*-*	*-*	*-*	0.113
	-	*-*	*-*	-	0.017–0.695	*-*	*-*	*-*	0.004–0.366	*-*	*-*	*-*	0.004–0.288

*P* denotes the predator density.

According to the Beddington-DeAngelis, Crowley-Martin and Hassell-Varley models, at the predator densities of one, two and three larvae, attack rates did not differ significantly among treatments. However, attack rate was significantly lower at the predator density of four larvae, compared to the other tested densities. Thus, attack rate decreased from 0.464h^-1^ in case of single predator to 0.208, 0.203 and 0.207 h^-1^ for the Beddington-DeAngelis, Crowley-Martin and Hassell-Varley models, respectively, when four predators were exposed. This trend was revealed and from the Holling type II model, although attack rate was marginally lower when two predators exposed together compared to the cases where one or three predators were examined. Also, the predator density did not affect the estimated magnitude of interference in each predator-dependent model, while zero was excluded from the estimated credible intervals.

The comparison of the tested models, based on the DIC criterion, revealed that Crowley-Martin model was the best in all three cases, followed by the Beddington-DeAngelis, Hassell-Varley, where the purely prey-dependent Holling type II model produced the worst fit (Table 2). Thus, the mechanistic predator-dependent Crowley-Martin model was consistently selected.

**Table 2:** Deviance information criterion for the Holling type II, Crowley-Martin, Beddington-DeAngelis and Hassell-Varley models, used for the model selection process. *P* denotes the predator density.

	DIC			
	*P = 1*	*P = 2*	*P = 3*	*P = 4*
Holling type II	266.677	355.676	531.900	657.865
Crowley-Martin		352.515	528.953	654.105
Beddington-DeAngelis		353.528	529.342	656.013
Hassell-Varley		354.021	530.556	657.104

## Discussion

Our study indicated that the feeding rate of an aphidophagous coccinellid at low prey densities is modified by a critical predator density. Also, when prey density becomes abundant, mutual interference has negligible effect upon the coccinellid’s larval instars feeding rate. We expect these results to have bearing on the use of the aphidophagous ladybirds as biocontrol agents. As the intensity of interference competition increases with coccinellid’s crowding at low prey densities, the predation efficiency of individuals is adversely affected. Absence of interference competition at high prey densities has also been reported for the coccidophagous ladybirds *Chilocorus bipustulatus* L., *C*. *nigritus* F. and *C*. *infernalis* Mulsant (Coleoptera: Coccinellidae) preying on *Aspidiotus nerii* Bouché (Homoptera: Diaspididae) [35], where there is no available information on their feeding behavior in lower prey densities. In addition, Agarwala et al. [36] reported that gravid females of the aphidophagous coccinellid *Menochilus sexmaculatus* Fabricious (Coleoptera: Coccinellidae) reduced oviposition, but not feeding, when exposed to immobilized conspecific. However, their experiments performed on a density of *A*. *craccivora* (Hemiptera: Aphididae) that the coccinellid could be satiated.

Our data suggest that *P*. *quatuordecimpunctata* handling time remained unaffected by predator density. This means that the time the larval instars spend on pursuing, subduing and eating their prey is unchanged by predator density. Papanikolaou et al. [37] suggest that *P*. *quatuordecimpunctata* is a digestive-limited predator, meaning that limitation of predation rate at high prey densities is attributed to satiation. It is expected that at high prey densities, the time lost during digestion breaks fully accommodates the cost of interference [38]. If digestion proceeds during interference interactions, the time cost of interference may be negligible [38]. On the other hand, we expect that at low prey densities digestion breaks are non-existent or negligible, so that above a critical predator density they waste notable time interfering with each other. This density may be determined by the relative size of predator and prey and/or the patch size. Patch in this sense means the space that the larva of a predator can reach by walking, usually one or a few adjacent plants, or even only a part of an individual plant. Thus, the functional response of a larva is determined by the situation encountered in the patch of prey it occupies [19, 20].

The differences between the DIC values for the different fitted models are fairly small. Based only on the DIC, the mechanistic approach of Crowley-Martin model appears to be slightly better than the other models, although the differences in DIC are fairly small. In general, there is no clear guidance upon what would constitute an important difference in DIC. However, Fig 1 reveals that the other models also offer a good fit to the data. On its initial derivation, Crawley & Martin used the fundamental process of predator’ feeding behavior presented in Beddington-DeAngelis model and extended its assumptions. According to Skalski and Gilliam [9], an important distinction between the Beddington-DeAngelis and Crowley-Martin models is that the Beddington-DeAngelis model assumes that the effects of predator’ interference on feeding rate becomes negligible under conditions of high prey abundance because predators that are handling prey do not interfere, whereas the Crowley-Martin model allows for interference competition among predators regardless of whether an individual is currently handling or searching for prey. Our data suggested that interference competition at high prey densities did not affect the feeding rate of the coccinellid. Thus, the time lost during prey handling is negligible. In addition, the model parameter *c*, which describes the magnitude of interference among predators, remained unaffected by the predator density. Nonetheless, its confidence intervals do not include zero indicating that this is a significant parameter. From a biological point of view, this constitutes further evidence that the predation of *P*. *quatuordecimpunctata* does not strictly follow the purely prey-dependent assumptions of Holling’s disc equation.

The application of functional response models that accounts for interference competition is underutilized so far in the ecological literature, partly due to their complexity. Fitting such models to experiments can be challenging due to issues of practical identifiability, which is when the experimental data do not provide enough information about the parameters of interest. In this paper we overcame such issues by taking advantage of the Bayesian framework and using informative priors for the parameter that is common across models.

In conclusion, laboratory experiments allowed us to provide inference in our twofold purpose, the viability and inference procedure of predator-dependent functional response models, as well as an indication of the mutual interference effect on the coccinellids’ feeding behavior in non-spatial scale. It could be interesting for future studies to consider interference competition in spatial sense [39, 40, 41]. In addition, our study is in line with Skalski and Gilliam [9] and Kratina et al. [10] suggesting that the predator-dependent functional response models that include effects of interference competition are viable and can provide additional insights regarding the predators’ feeding behavior, where no single functional response accurately describes predation. We expect that further field studies may provide useful information on intra-specific competition of aphidophagous ladybirds, accounting for environmental stochasticity. Moreover, studies on inter-specific interference or intraguild predation would be desirable in order to derive more information on coccinellids’ feeding behavior, as several models have been proposed in order to incorporate such interactions (e.g. [42, 43]).

## Supporting Information

S1 Table Raw data of the number of prey consumed (N_e_) at various initial prey densities (N_0_). P denotes the number of predators.
(PDF)
