# Supplementary material for: Does Mutual Interference Affect the Feeding Rate of Aphidophagous Coccinellids? A Modeling Perspective
Source: PLoS One. 2016 Jan 12;11(1):e0146168. doi: 10.1371/journal.pone.0146168 (PMC4710538; doi:10.1371/journal.pone.0146168)
Supplement: S1 Table — P denotes the number of predators. (PDF) [file pone.0146168.s001.pdf]

P=1		P=2		P=3		P=4	
N_0	N_e	N_0	N_e	N_0	N_e	N_0	N_e
5	5	10	10	15	15	20	19
5	4	10	10	15	15	20	20
5	5	10	10	15	15	20	20
5	5	10	10	15	15	20	19
5	5	10	9	15	11	20	20
5	4	10	5	15	13	20	18
5	4	10	10	15	15	20	19
5	4	10	8	15	15	20	20
5	5	10	9	15	15	20	18
5	5	10	7	15	15	20	19
10	9	20	20	30	29	40	33
10	10	20	18	30	28	40	38
10	4	20	16	30	29	40	40
10	9	20	16	30	29	40	26
10	7	20	19	30	30	40	31
10	6	20	20	30	24	40	38
10	8	20	20	30	28	40	39
10	9	20	19	30	30	40	40
10	8	20	18	30	23	40	40
10	10	20	18	30	27	40	28
15	12	30	18	45	40	60	56
15	15	30	14	45	44	60	40
15	10	30	24	45	43	60	38
15	14	30	21	45	41	60	55
15	13	30	30	45	44	60	59
15	12	30	26	45	45	60	40
15	15	30	14	45	44	60	48
15	10	30	23	45	39	60	40
15	12	30	20	45	30	60	35
15	14	30	25	45	35	60	32
20	17	40	26	60	55	80	74
20	20	40	30	60	60	80	44
20	13	40	26	60	55	80	78
20	12	40	29	60	31	80	76
20	12	40	36	60	46	80	77
20	15	40	30	60	26	80	68
20	20	40	32	60	22	80	49
20	11	40	40	60	39	80	52
20	8	40	24	60	50	80	45
20	9	40	20	60	57	80	38

25	18	50	40	75	48	100	86
25	25	50	32	75	45	100	72
25	17	50	25	75	53	100	45
25	10	50	24	75	27	100	39
25	16	50	23	75	23	100	61
25	24	50	30	75	30	100	52
25	11	50	13	75	26	100	35
25	21	50	34	75	29	100	42
25	11	50	24	75	53	100	34
25	18	50	41	75	60	100	46
